# Phytochemical evaluation, antioxidant assay, antibacterial activity and determination of cell viability (J774 and THP1 alpha cell lines) of P. sylvestris leaf crude and methanol purified fractions 

**DOI:** 10.17179/excli2015-689

**Published:** 2016-02-05

**Authors:** Dinesh C. Sharma, Ritu Shukla, Jasarat Ali, Swati Sharma, Priti Bajpai, Neelam Pathak

**Affiliations:** 1 Department of Biosciences, Integral University Lucknow, India-226026

**Keywords:** GC-MS, MPF, MTT assay, ROS

## Abstract

*Phoenix sylvestris* (Arecaceae family) known as Indian Date Palm has been identified as a component of traditional medicine against various ailments. The present study was focused on phytochemical screening of crude hexane, dichloromethane and methanol leaf extracts. The crude extracts showed the presence of alkaloids, flavonoids, and phenols in the plant leaves. In the study methanol extract was found most potent, so this extract was further fractionated by column chromatography and 9 methanol purified fractions (MPFs) were isolated. Most potential MPF8 (20:80 chloroform: methanol ratio fraction) significantly enhanced free radicals and antibacterial activity. The best MIC (Minimum inhibitory concentration) of MPF8 was investigated against *M. luteus* and *E. coli* at 1 mg/ml concentration. However, against other bacteria the MIC ranged from 1 mg/ml to 3 mg/ml. The GC-MS analysis showed the presence of many biologically active compounds such as alcohols, flavonoids, aromatic compounds, aldehydes, terpenoids fatty acid methyl esters, and phenolics. Pentadecanoic acid occupied maximum (52 %) area in GC-MS profiling. MPF8 was assayed for *in-vitro* cytotoxicity by MTT assay which confirms its less cytotoxicity at lower concentration and also significant ROS determination against J774 and THP1 cell lines after 2 and 4 hours.

## Introduction

The genus *Phoenix* L. is a Phoenicoid palm of Arecaceae family having 17 species found in diverse habitats, swamps, deserts and mangrove sea coasts (Zaid and Jimenez, 2002[26]; Page and Olds, 2004; Azmat et al, 2012[4]). Most *Phoenix* species originate in semi arid regions but usually occur at near high water levels, rivers or springs. The genus Phoenix is unique among the members of subfamily Coryphoideae being the only one with pinnate compound leaves (Corner, 1966[10]). *P. sylvestris* have also been reported for its traditional medicinal use by the tribal peoples (Chowdhury et al., 2010[9]; Gandhimathi and Sreedevi, 2012[11]; Salvi and Katewa, 2012[20]; Shailajam et al., 2012[21]) and also has been reported for its nutritive and protective activity. Previous studies have reported the different allergic responses of the pollen grains that result in inflammation of mucous membrane of nose (Chakraborty et al., 2006[8]). The biochemical analysis of pollens reported the presence of many phenolic compounds (Bhattacharya et al., 1993[5]) and they may be responsible for the allergic effects. 

Although all present new and better pharmacological medicines have been introduced to minimize the allergic responses, merely allergen immunotherapy targets the natural cause of allergic reaction (Bosquet et al., 1998[6]). The number of studies has been proven the efficacy of such therapy to allergic patients of airborne pollen grains (Walker et al., 1995[25]; Arvindson et al., 2002[2]; Moller et al., 2002[17]). *Phoenix sylvestris* may be a source of good immunomodulatory compounds with very low cytotoxicity as well as good antioxidant potential. Present study was carried out to compare potential methanol purified fraction for antioxidant potential, ROS generation and cytotoxicity. 

## Materials and Methods

### Materials

The different parts of *P. sylvestris *were collected from the vicinity of Integral University, Lucknow, Uttar Pradesh, India and authenticated by BSIP, Lucknow. The solvents and chemicals used in the study were of HPLC grade purchased from local source.

### Preparation of plant extract

**Figure 1:**
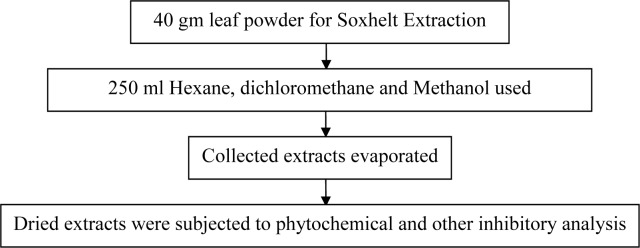
Schematic representation of extraction and further analysis

The collected leaves of *P. sylvestris *were cleaned with water and shade dried, and crushed in pestle-mortar to get a coarse powder (40 mesh size). This powdered material was processed for extraction by Soxhlet extraction continued for 8 cycles (6 hrs) using hexane, dichloromethane and methanol as a solvent. The extract was filtered and concentrated at reduced temperature on a rotary evaporator. Dried and weighed extract was stored at 4 °C (Figure 1(Fig. 1)).

### Phytochemical analysis 

All the extracts were qualitatively examined for phytochemicals by following the protocol of Adetuyi and Popoola (2001[1]), McDonald and co-workers (2001[16]), Trease and Evans (1989[24]) and Sofowora (1982[23]).

#### Tannins

Powdered materials (200 mg) were boiled in distilled water (10 ml) and add few drops of FeCl_3 _to the filtrate. After addition of FeCl_3_ a blue-black precipitation was indicated the presence of Tannins.

#### Alkaloids

200 mg powdered material was boiled in methanol (10 mL) and filtered. 1 % HCl was added followed by few drops of Dragendorff reagent. After addition prescribed reagents a brownish-red precipitate was shown the presence of alkaloids.

#### Flavonoids

200 mg of powdered material was boiled in distilled water and 5 ml of dilute ammonia solution was added to the filtrate followed by addition of concentrated H_2_SO_4 _(5 ml). An appearance of yellow colour confirmed the presence of flavonoids.

#### Saponins

200 mg of powdered material was homogenized with 5 mL distilled water. 0.5 ml filtrate has been taken and diluted with 5 ml of distilled water and shaken forcefully for 2 minutes. Appearance of stable foam was indicated the presence of saponins.

#### Phenols

Total phenols in plant samples were determined by the Folin-Ciocalteu method given by McDonald et al., (2001[16]). Samples with reaction mixture were incubated at room temperature for 15 minutes and absorbance at 765 nm was measured by using spectrophotometer. Total phenol values were expressed in terms of Gallic acid equivalent.

### Anti-oxidant studies 

All the crude extracts were screened for free radical scavenging activity by DPPH method and then further fractionated using column chromatography. All methanol purified fractions (MPFs) of the active extract were again examined for the 1,1-diphenyl-2-picrylhydrazyl (DPPH) and 2,2'-azino-bis(3-ethylbenzothiazoline-6-sulphonic acid) (ABTS) method of free radical scavenging activity. Following methods have been used for the examination of free radical scavenging potential (Re et al., 1999[19]; Ayoola et al., 2008[3]).

### DPPH radical scavenging assay

Free radical scavenging activity was determined by the 1,1-diphenyl-2-picrylhydrazyl (DPPH) radical assay. The DPPH 1 mM solution was freshly prepared in methanol and 3 ml of this solution was mixed with 100 μl of various concentrations (12.5, 25, 50, 100, 250, 500 µg/ml) of test samples. Ascorbic acid was used as reference. The samples were incubated for 30 min at 37 °C and absorbance was measured by spectrophotometre (Ependroff make) at 517 nm. The radical scavenging activity was calculated using the following formula: 

Percent inhibition = {[Ac-As] / Ac} x 100

where: Ac is the absorption of the blank sample; As is the absorption of the extract

### ABTS radical scavenging activity

ABTS (2,2'-azino-bis(3-ethylbenzothiazoline-6-sulphonic acid) free radical scavenging activity was analyzed by following standard protocol of Re and colleague (1999[19]). The ABTS cation radical was produced by the reaction between 5 ml of 14 mM ABTS solution and 5 ml of 4.9 mM potassium persulfate solution which was incubated for 16 hours in the dark at room temperature. Prior to use this solution was standardized by diluting on spectrophotometer at 734 nm to get an absorbance of 0.700 ± 0.020. The test sample at various concentrations (12.5, 25, 50, 100, 250, 500 µg/ml) with 1 ml of ABTS solution was homogenized and absorbance was recorded at 734 nm. Ethanol was used as a blank and all absorbance was taken within 6 min. The percentage inhibition was calculated using the formula: 

ABTS scavenging activity (%) = {[Ac-As] / Ac} x 100

where: Ac is the absorption of the blank sample; As is the absorption of the extract.

### Antibacterial activity

#### Preparation of bacterial culture

Pure cultures of ten test organisms, namely, *Micrococcus leuteus, Pseudomonas aeruginosa, Listeria monocytogens, Bacillus subtilis, Escherichia coli, Staphylococcus aureus, Staphylococcus epidermidis, Vibrio cholarae, Salmonella abony *and* Raoultella planticola* were purchased from National Chemical Laboratory, Pune. Stock cultures prepared on agar slants and stored at 4 °C. A loop full of microorganisms was transferred from stock culture to 50 mL of sterile nutrient broth for the preparation of working culture.

#### Well diffusion method

Antibacterial screening of crude extracts was carried out by well diffusion method on agar plates. 20 mL of sterile Mueller Hinton Agar media was poured in autoclaved Petri plates. After solidification of media, bacterial culture was swab on entire agar surface of each plate. The uniform wells were prepared by sterile 6mm diameter cork-borer. All well was filled with the different extracts (Hexane, dichloromethane and methanol) of 5 mg/ml (in DMSO) concentrations. The plates were then incubated at 37 °C for 24 hrs. The experiment was performed in triplicate and average zone of inhibition was calculated. As a negative control 9% DMSO was used. Bacterial culture was maintained at the turbidity of 1×10^8^ CFU/ml.

#### Determination of minimum inhibitory concentration

Minimum inhibitory concentration (MIC assay) of MPF (Islam et al., 2008[14]) has been evaluated against 10 pathogenic bacteria. Overnight incubated bacterial culture (turbidity equivalent to McFarland solution) having 1 to 2 ×10^8^ cfu/ml was used for the test. Different concentrations (0.1-5 mg/ml) of compound and drug (Rifampicine) were used for MIC determination.

#### Fractionation of the MPF

Silica (60-120 mesh) was mixed with petroleum benzene to make slurry and loaded in a 50 cm long and 2 cm width column. *Crude Leaf* methanolic extract was mixed with petroleum benzene and silica, which applied to the silica column. Different polarity solvents were prepared by using different Chloroform: Methanol ratio and different Methanol Purified fractions (MPF) have been eluted from column 1 ml/minute flow rate which were further analyzed for screening assays.

#### Gas chromatography mass spectroscopy analysis

The dried powder of the MPF8 of methanolic leaf extract was dissolved in the respective solvent and GC-MS analysis of sample was carried out by following process in the GC-MS machine. For the identification of compounds the samples were subjected to GC-MS analysis. The sample (1 μl) was injected into a RTX-5 column (60 m X 0.25 mm i.d., film thickness 0.25 μm) of GC-MS (model GC-MS-QP-2010 plus, Shimadzu). Helium was used as carrier gas and temperature programming was maintained from 100 °C to 200 °C with constant rise of 5 °C/min and then held isothermal at 200 °C for 6 min; further the temperature was increased by 10 °C/min up to 290 °C and again held iso-thermal at 290 °C for 10 min. The injector and ion source temperatures were 270 °C and 250 °C, respectively. Mass spectra were taken at 70eV, a scan interval of 0.5 °s and fragments from 40 to 950 Dalton. The final confirmation of constituents was made by computer matching of the mass spectra of observed peaks with the Wiley and National Institute Standard and Technology (NIST) libraries mass spectral database.

### Cell lines

Mouse macrophage cell line**s **(J774) and human macrophage cell line (THP1 α) were procured from the National Centre of Cell Sciences, Pune, India. Cells were maintained at Animal Tissue Culture (ATC) facility of Central Drug Research Institute (CDRI), Lucknow. Cell lines were preserved in Dulbecco's Eagle's medium (DEM) supplemented with 10 % FCS and 1 % antibiotic-antimycotic solution, 5 % CO_2_ using standard cell culture methods at 37 °C. 

### Cytotoxicity assay

MTT [3-(4,5-dimethylthiazol-2-yl)-2,5-diphenyltetrazolium bromide] conversion assay was carried out for the determination of cell viability. There were 1 x10^6^ cells/ml plated in 96-well culture plates which than incubated with increasing concentrations of MPF8 (2, 5, 10, 25, 50 and 100 μg/ml) for 24 hr in CO_2_ incubator at 37 °C. The dye (MTT) was added to each well and plate was incubated at 37 °C for 4 hr. The intensity of formazan was measured by spectrophotometer Powerwave XS, BIOTEK, USA at 550. The percent of cell viability was calculated by following formula:

Cell viability (%) = Mean OD / control OD × 100

### Intracellular reactive oxygen species measurement

Intracellular oxidative stress was calculated with the help of (DCFH-DA) 2',7'-dichlorofluorescin di-acetate (Cathcart et al., 1984[7]). The experiment was carried out as described earlier by Goswami and co-workers (2016[12]) with slight modifications in our lab. Primarily, confluent cells were seeded (1000 cells/well) into black bottomed plates (96 well) and prior to exposure cells were allowed to adhere for a period of 24 hrs. For ROS quantification, both cells (mouse and human macrophage) were plated and distributed in triplicate manner. A working stock of 20 µM DCFH-DA was prepared in PBS and all the test concentrations of MPF8, negative controls and positive controls were prepared which were exposed to the cells in this working stock. The negative control contained of the working stock solely a 20 µM DCFH-DA solution in PBS, the positive control consisted of 1 µM Hydrogen Peroxide (H_2_O_2_) in 20 µM DCFH-DA/PBS working stock solution and finally the test concentrations of isolated compounds. For the stimulation of macrophages lipopolysaccharide (1 µg/ml) was used as a mitogen. 10 µg/ml test concentration for compound and the incubation period ranged from 2 hr, 4 hr and 6 hr. The rate of intracellular oxidative stress was observed by measuring their fluorescence intensity via fluorometer (BIOTEK-FLX800'USA) emission at 520 nm (by 485 nm excitation).

### Statistical analysis 

All the experiments were performed in triplicate manner and results were expressed as mean ± SD. Statistically significant differences were set at p < 0.01. 

## Results

### Phytochemical analysis

The present study was carried on hexane, dichloromethane and methanolic extracts of *Phoenix sylvestris *leaf to find out the presence of medicinally important phytocompounds. All the crude extracts and MPFs revealed the presence of various phytochemicals such as tannins, saponins, flavonoids, alkaloids and phenols (Table 1(Tab. 1)).

### Antioxidant activity

**Figure 2:**
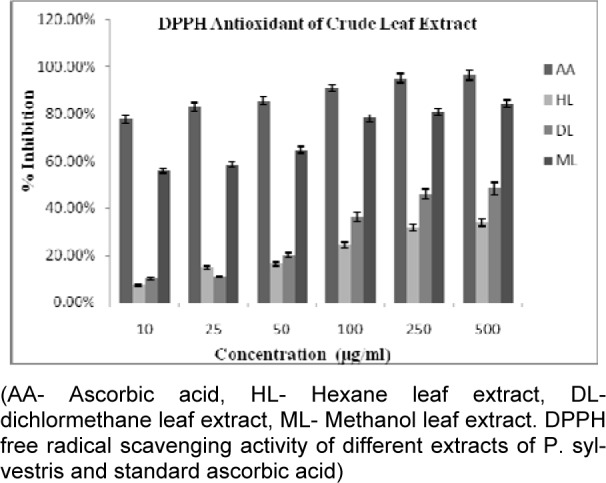
Antioxidant activity of *P. sylvestris* crude leaf extracts

**Figure 3:**
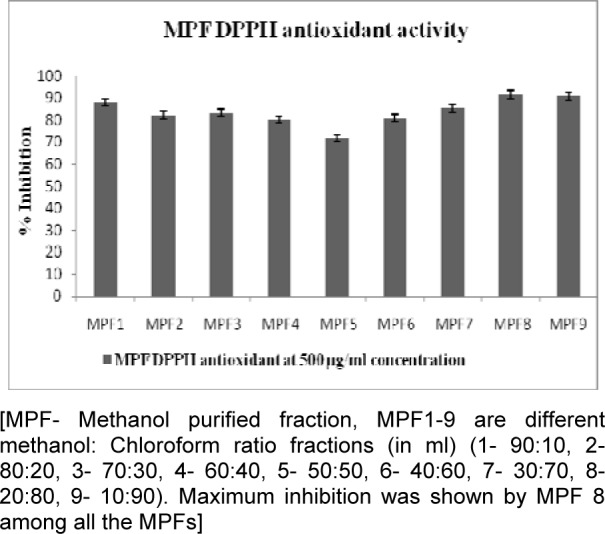
Antioxidant activity of *P. sylvestris* MPFs extracts

**Figure 4:**
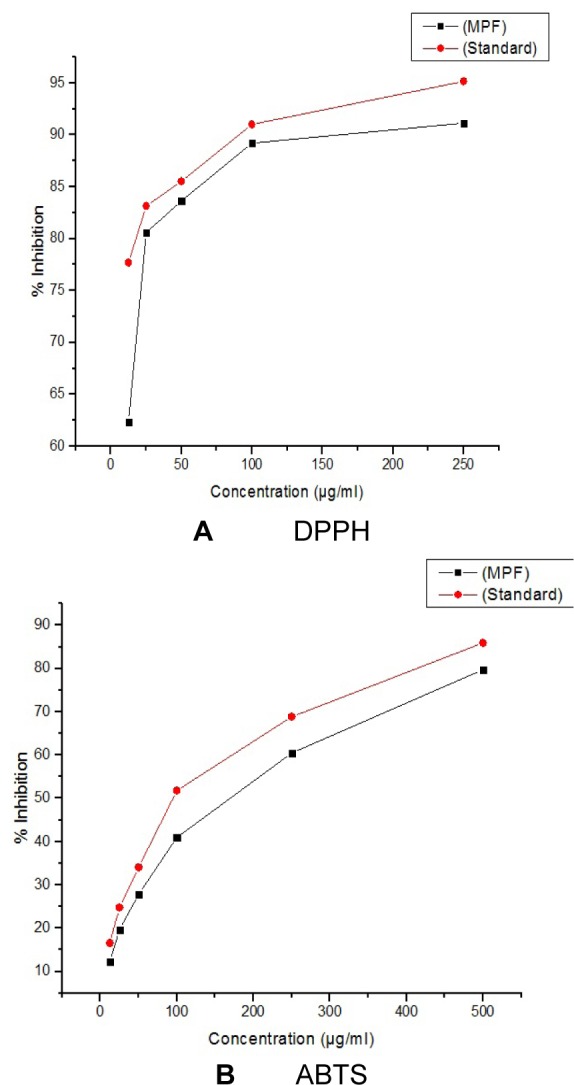
A (DPPH) and B (ABTS): Antioxidant activity of P. sylvestris MPF8 extract (P. sylvestris MPF8 extract referencing the standard ascorbic acid value in DPPH and ABTS methods)

All crude extracts of leaf have been examined for antioxidant activity by DPPH method and methanol extract was found to inhibit maximum percent of free radicals (84.35 %) at 500 µg/ml concentration. This potential extract was further fractionated by column chromatography and 9 MPFs collected, were assayed for their different free radical scavenging activity. MPF8 at maximum concentration (250 µg/ml), 91.14 % and 79.73 % free radical inhibition was observed by DPPH assay and ABTS assay respectively where as standard compound ascorbic acid showed 95.16 % (DPPH) and 85.90 % (ABTS) free radical inhibition (Figure 2(Fig. 2), 3(Fig. 3) and 4(Fig. 4)). 

### Antibacterial study

**Figure 5:**
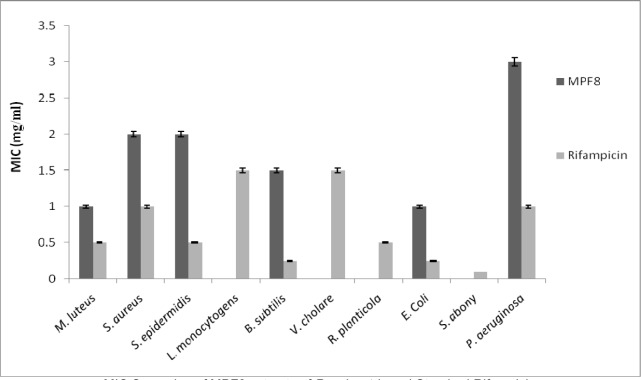
Minimum Inhibitory Concentration determination of P. sylvestris MPF8 extract

The present investigation showed the antibacterial efficacy of all the extracts against the selected pathogenic bacteria (Table 2(Tab. 2) and Figure 5(Fig. 5)). The methanolic extract showed highest antibacterial activity against *B. subtilis *(2.2 cm) followed by *E. coli* (2.1 cm) and *P. aeruginosa *(2.0 cm)*. *The methanol extract was most potent against *B. subtilis*, showing the maximum inhibition zone at the concentration of 5 mg/ml. The highest sensitivity of *B. subtilis* may be due to its cell wall constitution and outer membrane. This methanolic extract was fractionated and MPF8 was screened as a most potent fraction on the basis of antioxidant activity. MPF8 was evaluated for MIC determination against pathogenic bacteria and best MIC value was reported at 1mg/ml concentration against *M. luteus *and *E.coli *followed by followed by 1.5 mg/ml, 2 mg/ml against *B. subtilis, S. epidermidis and S. aureus. *Rifampicine was used as standard drug which inhibited bacteria significantly. 

### Gas chromatography-mass spectroscopy analysis

**Figure 6:**
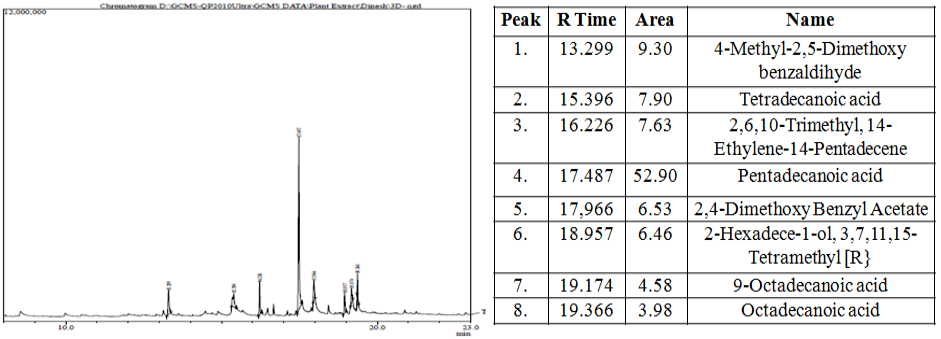
Showing GC-MS compounds peaks and their area of *P. sylvestris* MPF8 extract

The most potent MPF8 was analyzed by GC-MS method and revealed the presence of eight compounds 4-Methyl-2,5-Dimethoxybenzadihyde, Tetradecanoic acid, 2,6,10-Trimethyl,14-ethylene-14-pentadecene, Pentadecanoic acid, 2,4-Dimethoxybenzyl acetate, 2-hexadecen-1-ol, 3,7,11,15-Tetramethyl, 9-Octadecanoic acid. Their percent area and respective retention time is shown in Figure 6(Fig. 6). Antioxidant, antibacterial and other activity were due to the synergistic effect of all above compounds. Pentadecanoic acid demonstrated maximum percent area (52.90) in the GC-MS analysis and was previously identified as strong anti-inflammatory, antibacterial and anti-cancerous activity where as other compounds identified in GC-MS study, possess significant protective and curing property (Figure 6(Fig. 6)). 

### Cell viability and ROS assay

**Figure 7:**
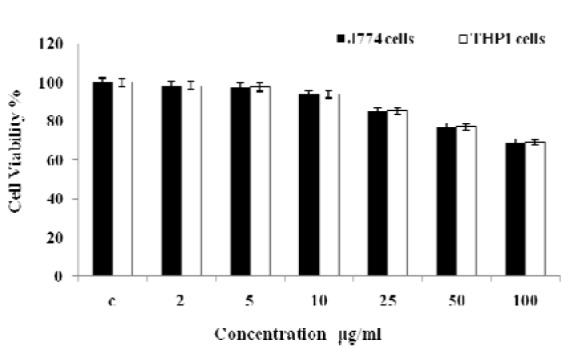
Cell viability study of *P. sylvestris* MPF8 extract (MTT assay of J774 and THP1 α cell line)

Cell viability has been evaluated by MTT assay against J774 and THP1 cell lines (Figure 7(Fig. 7)). MPF8 showed no cytotoxicity at lower concentration (2-10 µg/ml) and at higher concentration (100 µg/ml), it showed significant cell viability in comparison to control. 

**Figure 8:**
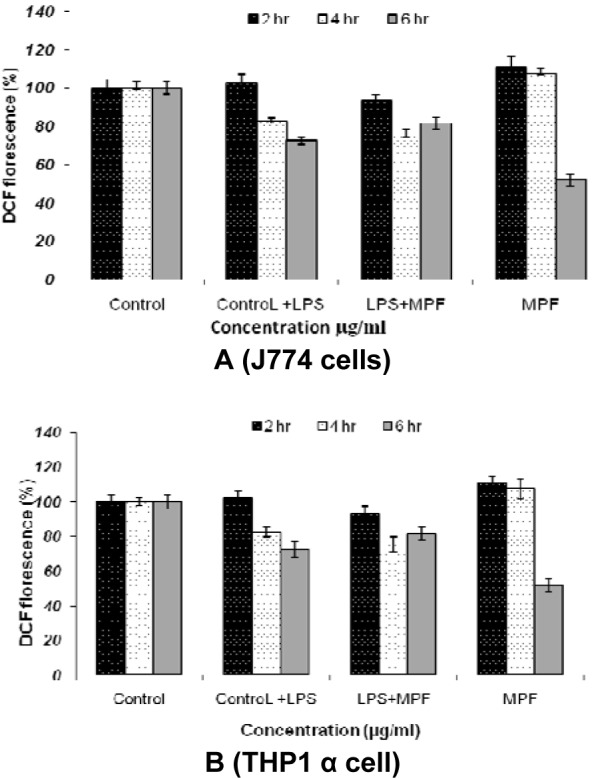
ROS estimation in J774 cell lines (A) and THP1 α cell lines (B) after incubation with *P. sylvestris* MPF8 extract at various time intervals (2 h, 4 h, 6 h)

2',7'-dichlorofluorescein emission by the macrophage cell lines which was incubated for various time intervals demonstrated that MPF8 was able to generate significant ROS activity from both the cell lines indicating the macrophage stimulation. The best results were obtained after 2 and 4 hr incubation, which declined at 6 hrs (Figure 8(Fig. 8)). 

## Discussion

*P. sylvestris* has been known since ancient time for its nutritive and medicinal properties as hepato-protective, anti-inflammatory, anti-pyretic etc (Howlader et al., 2006[13]; Chowdhury et al., 2010[9]; Gandhimathi and Sreedevi, 2012[11]; Shailajam et al., 2012[21]). During the present investigation, best and potential methanolic leaf extract of plant was fractionated by using silica gel column. In order to find out potential MPF, all fractions were screened on the basis of *in-vitro* antioxidant analysis. MPF8 was screened and significant free radical scavenging activity was found, thus, it may be used to minimize diabetes and cancerous condition without causing any toxic effects. The phytochemical study revealed the presence of several compounds which are individually reported for immunomodulatory, anti-microbiial, anti-inflammatory, anti-cancerous and other protective studies (Kothari, 2011[15]; Nag et al., 2012[18]; Shukla et al., 2015[22]). Significant antibacterial and antioxidant activity may be due to the synergistic effect of these compounds. 

Natural compounds are best for the treatment because of their low toxic effect as observed during this study. MPF have significant protective activity showing negligible toxicity at higher concentration which was little more in THP1 α cells as compared to J774 which can be recognized to the natural anticancer property of MPF. The present study demonstrated that the higher doses of MPF produced cytotoxicity on both the cell lines. The GC-MS analysis of MPF revealed the presence of several phenolic compounds which can be suggested as immuno-modulatory against the allergic response (Chakraborty et al., 2006[8]). This MPF may be more effective against the bacterial infections as was observed during this study. 

Our analysis found MPF8 as a significant antioxidant and ROS generating fraction possessing low cytotoxicity. This compound may be used for immune activation by immunomodulation.

## Conclusion

MPF8 was an isolated fraction from Methanol leaf extract that has significant phenolic compounds possessing antioxidant activity, antibacterial activity, and remarkable cell viability with significant ROS generation in comparison to crude extract. Our studies also suggested lower cytotoxicity of MPF8 against J774 and THP1 α cell line which promises its immunotherapy responses. Present study concludes that this medicinal plant have diversity of curing properties as well as immune-modulatory activity which is still not investigated and may be helpful for the treatment of various susceptible abnormalities of human being with improved clinical and immunologic outcome. 

## Acknowledgement

All authors would like to acknowledge Prof. S. W. Akhter, Hon`ble Vice chancellor of Integral University, Lucknow for providing laboratory facilities for the research work.

## Conflict of interest

The authors declare no conflict of interest.

## Figures and Tables

**Table 1:**
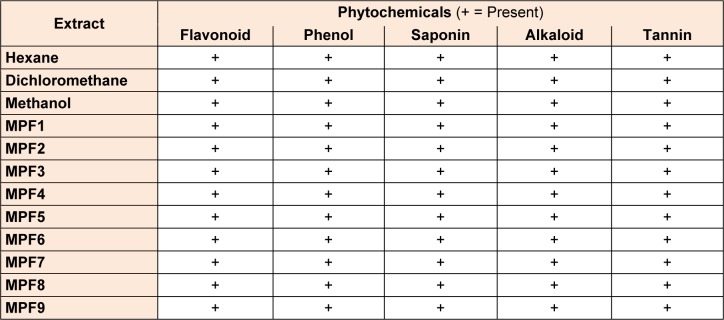
Phytochemical constituents of crude extracts and MPFs

**Table 2:**
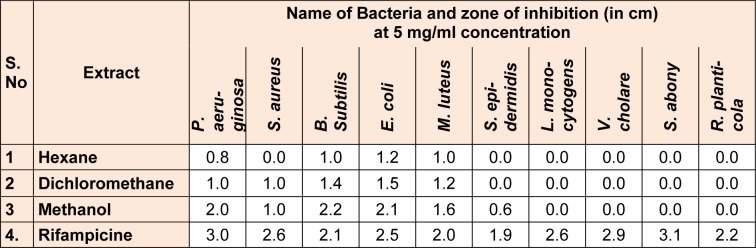
Antibacterial activity of *P. sylvestris *leaf crude extracts
